# 

**Published:** 1889-02

**Authors:** 


THE BUFFALO MEDICAL AND SURGICAL ASSOCIATION will hold its regular monthly meeting on Tuesday evening, February 12, 1889, at the Mechanics' Institute, No. 9 West Mohawk street. Subject: "Syphilis; Its Later Manifestations and Its Curability." Discussion by Dr Charles G. Stockton: "Medical, including Nervous." Dr. Roswell Park: "Surgical." Dr. William H. Heath: "Genito-Urinary." Dr. Elmer G. Starr: "Eye and Ear." Dr. F. H. Potter: "Throat." Dr. Ernest Wende: "Skin;" and Dr. M. D. Mann: "Heredity."

New Instrument.—An ingenious and useful instrument, combining cupping apparatus, breast-pump, aspirator, and stomach-pump, has been devised by Drs. Black, of Canada. The price is such as to bring it within the reach of the majority of physicians who need to be prepared with such instruments, yet have only an occasional necessity for their use.

---

Ready early in February.—The Transactions of the American Association of Obstetricians and Gynecologists for 1888. One handsome octavo volume, containing nearly 500 pages, profusely illustrated, and bound in cloth, with red leather back. Price, $5.00, including postage. Orders should be sent in at once to

William Warren Potter, M. D., *Secretary*,
284 Franklin street, Buffalo, N. Y.

---

The Journal of Cutaneous and Genito-Urinary Diseases has passed from its former publishers, Messrs. William Wood & Co., to the hands of Messrs. D. Appleton & Co. In the editorial management, Dr. Prince A. Morrow, who has heretofore so ably conducted this valuable journal, has associated with him Dr. John A. Fordyce—names that fully assure its continued instructive excellence.

---

Messrs. J. B. Lippincott Company announce to the profession the publication of a Cyclopedia of the Diseases of Children, medical and surgical, by American, British, and Canadian authors, edited by John M. Keating, M. D., in four imperial octavo volumes; to be sold by subscription only. The first volume will be issued early in April, and the subsequent volumes at short intervals.

A thorough knowledge of the diseases of children is a matter of the greatest importance to most physicians, and as this is the only work of the kind that has been published in English, it will be invaluable as a text-book and work of reference for the busy practitioner.

### BOOKS RECEIVED.

Thomas Jefferson and the University of Virginia. By Herbert B. Adams, Ph.D., Associate Professor of History in the Johns Hopkins University. U. S. Bureau of Education. Washington: Government Printing Office. 1888.

The Physicians' Leisure Library. Diseases of the Kidneys. By Dujardin-Beaumetz, M.D. Detroit: Geo. S. Davis. 1888.

Clinical Lectures on Albuminuria. By Thomas Grainger Stewart, M. D.; Professor of the Practice of Physic and Clinical Medicine in the University of Edinburgh. New York: Wm. Wood & Co. 1888.

The Pathology and Treatment of Displacements of the Uterus. By Dr B. S. Schultze, Professor of Gynecology, Director of the Lying-in Institution, and of the Gynecological Clinic, in Jena. Translated from the German by Jameson J. Macan, M.A., M.R.C.S., Eng., etc., and edited by Arthur V. Macan, M.B., M.Ch., etc., Master of the Rotunda Hospital, Dublin. With one hundred and twenty illustrations. Pp. 378. New York: D. Appleton & Co. 1888.

Archives de Physiologie Normale et Pathologique. Directeur, M. Brown-Séquard; Sous-Directeurs, M. M. Dartre, Professeur à la Faculté des Sciences, et François-Franck, Membre de l'Académie de Médicine. Cinquème série. Tome Premier. Nos. 1, 2. Janvier et Avril. 1889. Avec 2 Planches et 58 Figures dans le texte. Paris: G. Masson, Editeur, 120 Boulevard Saint Germain.

A Practical Treatise on Nervous Exhaustion (Neurasthenia): Its Symptoms, Nature, Sequences, and Treatment. By George M. Beard, A. M., M. D. Edited, with notes and additions, by A. D. Rockwell, A. M., M. D., Professor of Electro-Therapeutics in the New York Post-Graduate Medical School and Hospital; Fellow of the New York Academy; Member of the American Neurological Association; of the New York Neurological Society, etc. New York: E. B. Treat, 771 Broadway. 1889. Medical classics. Price, $2.75.

Wood's Medical and Surgical Monographs. Vol. I., No. 2. February, 1889. Contents: Gonorrheal Infection in Women, by William Jap Sinclair, A. M., M. D.; On Giddiness, by Thomas Grainger Stewart, M. D.; Albuminaria in Bright's Disease, by Dr. Perrie Jaenton. New York: William Wood & Co. 1889.

Transactions of the Association of American Physicians. Third Session; held in Washington, D. C., September 18, 19 and 20, 1888. Philadelphia. 1888.

### PAMPHLETS RECEIVED.

Report of the Surgeon-General of the Army to the Secretary of War, for the Fiscal Year ending June 30, 1888. Washington: Record and Pension Division Surgeon-General's Office. 1888.

Industrial Education in the South. By Rev. A. D. Mayo. Bureau of Education, Circular of Information No. 5, 1888. Washington: Government Printing Office. 1888.

Cornell University. College of Agriculture. Bulletin of the Agricultural Experiment Station III. November, 1888. I. The Insectary of Cornell University. II. On Preventing the Ravages of Wire-Worms. III. On the Destruction of the Plum Curculio by Poisons. Published by the University, Ithaca, N. Y. 1888.

The Philosophy of Memory. By D. T. Smith, M. D., Lecturer on Medical Jurisprudence, University of Louisville. Reprint from the *Practitioner and News.* Louisville: John P. Morton & Co. 1888.

The Surgical Advantages of the Buried Animal Suture, and its Adaptability to Special Purposes. By Henry O. Marcy, A. M., M. D., LL. D., Boston, Mass.

Read in the Section on Surgery, at the thirty-ninth annual meeting of the American Medical Association, May, 1888. Reprint from the *Journal of the American Medical Association*, July 21, 1888.

Also, by the same author, Placental Development. Reprint from the Transactions of the Ninth International Medical Congress. Vol. II.

Also, by the same author, The Histology and Surgical Treatment of Uterine Myoma. Reprint from the Transactions of the Ninth International Medical Congress. Vol. II.

Also, by the same author, The Climate of the Southern Appalachians. Reprint from the Transactions of the Ninth International Medical Congress. Vol. V.

I. Below Sea-Level. Nature's Pneumatic Cabinet. II. High Altitudes of Southern California. By Walter Lindley, M. D., Los Angeles, Cal. Reprint from the *Southern California Practitioner*, 1888.

The Means of Effecting the Unity of the Medical Profession. Being the Anniversary Discourse delivered before the New York Academy of Medicine, Thursday evening, November 15, 1888. By D. B. St. John Roosa, M. D., LL. D. New York: Trow's Printing and Bookbinding Company, 201–213 East Twelfth street. 1888.

Naso-Pharyngeal Fibromata. By E. Fletcher Ingals, A. M., M. D., Professor of Laryngology, Rush Medical College; Professor of Diseases of the Throat and Chest, Women's Medical College, Chicago. Reprint from the *Journal of the American Medical Association*, December 8, 1888.

Forced Respiration. History—Report of Six Cases; Apparatus; Effects of, on Narcotised Human Subjects; Adaptability of, in cases of Drowning or Shock. By George E. Fell, M. D., Buffalo, N. Y., Professor of Physiology and Microscopy, Medical Department of Niagara University. Reprint from Transactions of the New York State Medical Association for 1888.

Twentieth Annual Report of the Trustees of the Willard Asylum for the Insane, for the Year 1888. The Troy Press Company, Printers, 1889.

Pulmonary Consumption Considered as a Neurosis. Being two of a series of evening lectures given by the Faculty of the Philadelphia Polyclinic in the course of 1888 and 1889. By Thos. J. Mays, M. D., Professor of Diseases of the Chest in the Philadelphia Polyclinic. Reprint from the *Therapeutic Gazette*, November 15 and December 15, 1888.

The Wrong of Craniotomy upon the Living Fetus. Sixth Annual Address of the President, delivered before the Washington Obstetrical and Gynecological Society, October 19, 1888. By Samuel C. Busey, M. D., Washington, D. C. Reprint from the *American Journal of Obstetrics and Diseases of Women and Children*, Vol. XXII., January, 1888.

Annual Report of the Rhode Island Hospital, for 1888.

The Ingleside, Christmas Number. For 1888. Almanac for 1889. Published by the United States Life Insurance Company. New York: 261 Broadway.

The "Don't Forget It" Calendar, Daily Record, and Blotter. E. B. Treat, publisher, 771 Broadway, New York.

### HEALTH REPORTS.

State Board of Health Bulletin. Tennessee. Nashville, December 15, 1888.

Health in Michigan. December, 1888.

Monthly Bulletin of the New York State Board of Health. November, 1888.

Reports of Deaths and Contagious Diseases in Newport, R. I. December, 1888.

Weekly Abstract of Sanitary Reports U. S. Marine Hospital Service. Vol. III., Abstract No. 52. Vol. IV., Abstracts Nos. 1, 2, and 3.

